# CASK Positively Regulates Keratinocyte Proliferation and Modulates the EGFR-ERK-p21 Signaling Axis

**DOI:** 10.3390/ijms27219909

**Published:** 2026-11-05

**Authors:** Yi-Xuan Wang, Duen-Yi Huang, Keith Jun Hao Cheong, Ching-Yuan Cheng, Hua-Ching Chang, Wan-Wan Lin

**Affiliations:** 1 Department of Pharmacology, College of Medicine, National Taiwan University, Taipei 100, Taiwan; d04443005@ntu.edu.tw (Y.-X.W.); tyh123kimo@gmail.com (D.-Y.H.); r10443021@ntu.edu.tw (K.J.H.C.); chingyuancheng@ntu.edu.tw (C.-Y.C.); hc.chang@tmu.edu.tw (H.-C.C.); 2 Department of Dermatology, School of Medicine, College of Medicine, Taipei Medical University, No. 250, Wuxing Street, Xinyi District, Taipei 110, Taiwan; 3 Graduate Institute of Medical Sciences, Taipei Medical University, Taipei 110, Taiwan

**Keywords:** CASK, keratinocyte, EGFR, ERK, p21, p53

## Abstract

CASK is a multidomain scaffold protein with diverse cellular functions, but its role in keratinocytes remains unclear. In this study, we established a CASK-silenced human HaCaT keratinocyte model to investigate its biological function. We found that CASK was localized to the plasma membrane, cytosol, and nucleus, and its silencing suppressed keratinocyte proliferation and induced G1-phase arrest. RNA sequencing revealed that CASK depletion attenuates proliferation-associated transcriptional programs. Consistent with this, CASK silencing increased p53 and p21 expression while reducing cyclin B and phosphorylated Rb levels, with elevated p53 and p21 mRNA in shCASK cells. However, CASK silencing-associated p21 upregulation is not majorly dependent on p53 activity. Mechanistically, CASK silencing enhanced serum-induced activation of EGFR and downstream ERK signaling, which were accompanied by increased expression of the EGFR ligands amphiregulin, TGF-α, and epiregulin, indicating augmented autocrine/paracrine EGFR signaling. Pharmacological inhibition studies demonstrated that activation of the EGFR–ERK signaling pathway is essential for p21 upregulation upon CASK silencing. Promoter reporter analyses further revealed that CASK deficiency enhances p21 transcription. Collectively, these findings reveal a previously unrecognized role of CASK in regulating keratinocyte growth and cell-cycle progression. CASK promotes keratinocyte proliferation by restraining autocrine/paracrine EGFR–ERK signaling and suppressing p21 expression, thereby contributing to epidermal homeostasis and growth control.

## 1. Introduction

Calcium/calmodulin-dependent serine protein kinase (CASK), also known as LIN-2, is an evolutionarily conserved X-linked gene that encodes a scaffold protein belonging to the membrane-associated guanylate kinase (MAGUK) family [1]. CASK comprises several protein–protein interaction domains, including an N-terminal CaMK domain, L27 domain, PDZ domain, SH3 domain, and a C-terminal guanylate kinase (GUK) domain [2]. Although the GUK domain structurally resembles an active guanylate kinase, it is generally considered a catalytically inactive pseudokinase that lacks the ability to mediate phosphotransfer from ATP to GMP [3]. In contrast, the enzymatic activity of the CaMK domain remains incompletely understood, and only a limited number of substrates have been identified to date, including neurexin [4,5], α-synuclein [6], and AKT [7]. Nevertheless, owing to its highly conserved nature and multifunctional scaffolding properties, CASK has attracted considerable interest and has been extensively investigated in diverse tissues and biological systems in recent years [8,9].

CASK is ubiquitously expressed across multiple organs and tissues, with particularly high levels observed in the brain [10]. At the subcellular level, CASK localizes to the cytoplasm, plasma membrane, and nucleus, reflecting its diverse biological functions [11]. Compared with other tissues, researchers have characterized CASK’s physiological roles and regulatory mechanisms most extensively in the nervous system, where it contributes to synaptic transmission, neuronal differentiation, and neural development [12,13]. Mutations in the CASK gene are predominantly associated with neurodevelopmental disorders, including intellectual disability, microcephaly, seizures, and hypotonia [8,12,14,15,16]. Heterozygous CASK-deficient (CASK^+/−^) female mice exhibit postnatal microcephaly, cerebellar and optic nerve hypoplasia, and locomotor abnormalities [17]. The functions of CASK in synaptic plasticity and brain development are largely attributed to its scaffolding activity, which enables interactions with numerous presynaptic and postsynaptic proteins [4,18].

EGFR signaling plays a pivotal role in maintaining epidermal homeostasis by balancing keratinocyte proliferation and differentiation. Sustained activation of EGFR promotes keratinocyte growth and cell cycle progression, whereas attenuation or modulation of this pathway can shift cellular programs toward differentiation [19,20]. Among the downstream effectors governing these processes, p21 functions as a critical regulator of cell cycle progression and serves as a key mediator of keratinocyte growth control [21,22]. In this aspect, the EGFR–ERK signaling has been shown to increase p21 mRNA expression in various cell types, including keratinocytes [22,23,24,25,26,27,28,29]. Moreover, a study in *C. elegans* highlighted CASK’s role in EGFR trafficking. In *C. elegans*, CASK (LIN-2) facilitates the localization of EGFR (LET-23) to the basal membrane domain of vulval precursor cells, thereby promoting activation of the EGFR/Ras/MAPK signaling cascade required for vulval cell differentiation [30]. In the present study, we investigated the role of CASK in keratinocyte proliferation and the molecular mechanisms underlying EGFR signaling. Our findings demonstrate that CASK acts as a positive regulator of keratinocyte growth by suppressing p21 expression through the decreased EGFR–ERK signaling axis.

## 2. Results

### 2.1. CASK Silencing Attenuates Transcriptomic Expression of Proliferative and DNA Repair Programs in HaCaT Cells

**Figure 1:**
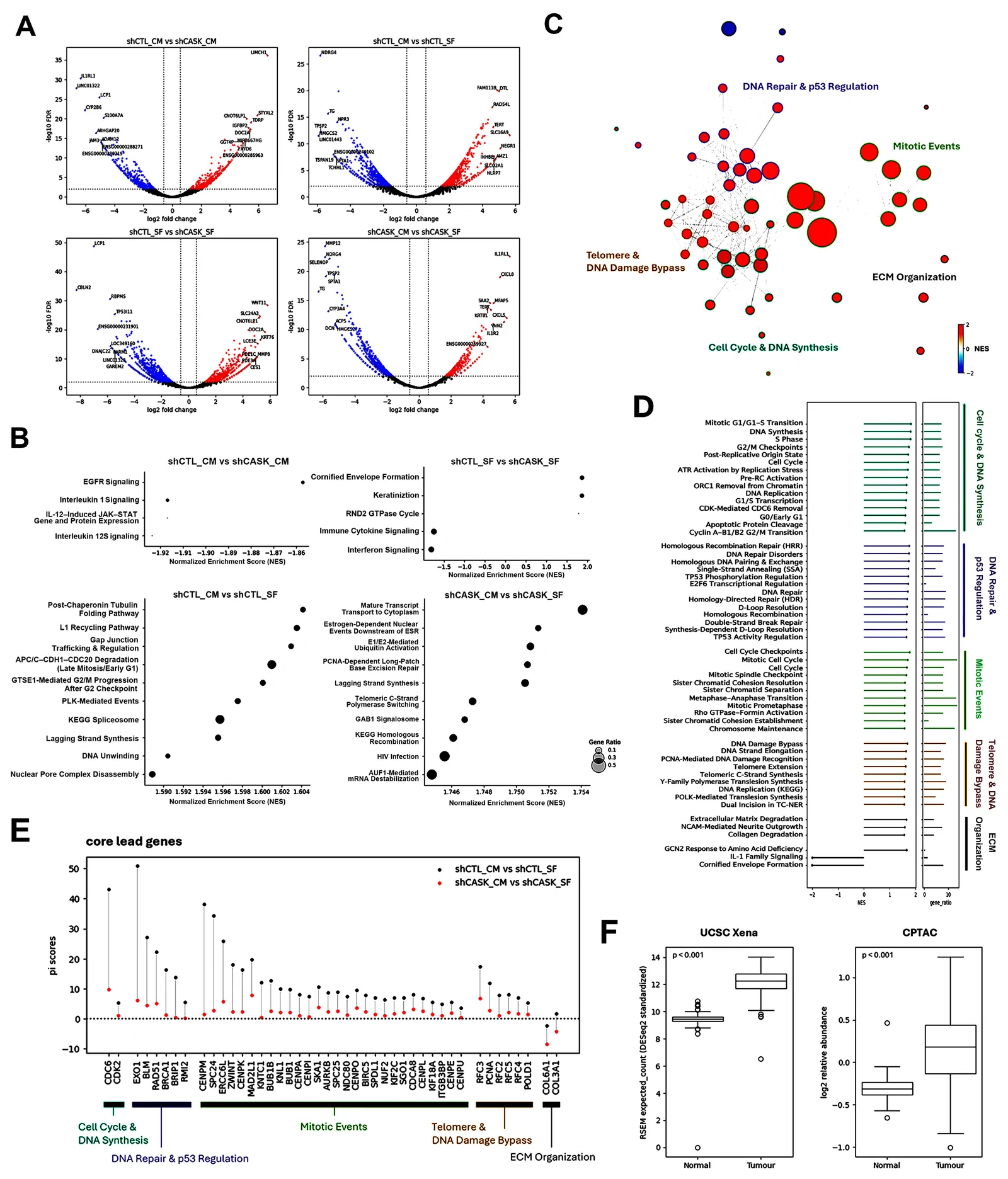
**CASK enhances the induction of proliferative programs upon serum restoration.** (**A**) Volcano plot shows differential expression comparing serum response (CM vs. SF in shCTL or shCASK cells) and CASK silencing in fixed conditions (shCTL vs. shCASK cells in complete medium or serum-free conditions), and (**B**) enriched pathways from GSEA. (**C**) Pathway enrichment network constructed from ΔΔπ scores computed from control vs. shCASK difference in expression gain from serum-free to complete medium, and (**D**) its corresponding lollipop plot detailing Normalized Enrichment Scores and gene ratio of each involved pathway. (**E**) “Core lead genes” were identified with degree centrality > 0.8 relative to the total number of pathways within their respective cluster, with a connected dot plot showing their serum restoration π-scores in shCTL and shCASK cells. (**F**) Differential expression of CASK was computed between normal and tumour LUSC tissues in transcriptomic (UCSC Xena) and proteomic (CPTAC) datasets.

To investigate the role of CASK in keratinocytes’ proliferation, we silenced CASK expression in HaCaT keratinocytes using lentiviral shRNA (shCASK). We utilized RNA-Seq and ΔΔπ score analysis to characterize the global transcriptomic shift in HaCaT cells upon the transition from serum-free to complete medium conditions and determine the involvement of CASK in both processes. As shown in the volcano plots, differential gene expression under steady-state conditions, cultured in either serum-free or complete medium, was observed in both control and CASK-silencing cells (Figure 1A). Interestingly, GSEA demonstrated that EGFR signaling-related pathways were negatively enriched in control cells compared with shCASK cells, indicating that EGFR-associated signaling may contribute to the regulation of serum-induced biological responses (Figure 1B, upper left panel). When comparing CASK-silenced and control HaCaT cells under serum-free conditions, the differentially enriched pathways were primarily associated with immune signaling (Figure 1B, upper right panel). Furthermore, GSEA also revealed significant positive enrichment of cell cycle-related pathways in control cells after the transition from serum-free to complete medium conditions (Figure 1B, lower left panel). Among these, the most enriched pathway was mitotic progression and early G1 transition, accompanied by CDC20 degradation and enrichment of additional proliferative pathways for G2/M phase progression, such as GTSE1, PLK-mediated events, and DNA unwinding processes. In contrast, no significant enrichment of cell cycle-related pathways was detected in shCASK cells during the transition from serum-free to complete medium (Figure 1B, lower right panel), suggesting that CASK silencing impairs serum-induced activation of proliferative signaling in keratinocytes.

Additionally, ΔΔπ score analysis comparing control and CASK-silenced cells during the serum response revealed that the induction of proliferative and DNA repair programs following the serum-free to complete medium transition was markedly enhanced in control cells relative to shCASK cells (Figure 1C,D). Among the cell cycle and DNA synthesis categories, pathways associated with mitotic G1/S transition, DNA synthesis, S phase progression, and G2/M checkpoint regulation represented the four most highly enriched pathways (Figure 1D). Similarly, within the DNA repair and p53 regulation categories, the highest enrichment scores were predominantly associated with homologous recombination-mediated DNA repair, DNA strand exchange, and annealing processes. Furthermore, pathways related to cell cycle checkpoints, mitotic progression, and spindle assembly checkpoint regulation were also among the top-enriched signatures within the mitotic events category (Figure 1D). These data indicate that CASK strengthens the magnitude of the proliferative gain upon serum restoration and highlights a regulatory role for CASK in keratinocyte proliferation. 

To pinpoint the molecular drivers of this effect, we identified “core lead genes” within each pathway cluster and compared the serum restoration π-scores of the core lead genes in control and CASK-silenced cells. In all instances, the π-scores were higher in the control cells (Figure 1E), confirming that CASK depletion attenuates the induction of those core genes. Within the cell cycle and DNA synthesis pathway cluster, CDC6 and CDK2 were highly enriched, and the coordinated activity of these two genes has been widely reported to promote the G1–S phase transition. Exonuclease 1 (EXO1), Bloom syndrome protein (BLM), and RAD51 were among the most highly enriched genes within the DNA repair and p53 regulation pathway cluster. These three genes are well known for maintaining genomic stability through distinct mechanisms during the S and G2 phases of the cell cycle. Within the mitotic events pathway cluster, Centromere Protein M (CENPM), SPC24, a component of the NDC80 kinetochore complex (SPC24), and ERCC excision repair 6-like (ERCC6L) were among the top enriched genes. Previous studies have reported that these genes play critical roles in chromosome segregation and spindle assembly checkpoint regulation during the M phase. Finally, to evaluate the clinical relevance of this CASK-mediated regulation, we examined CASK expression in keratinocyte-derived cancers such as LUSC (Figure 1F). We found that CASK is significantly overexpressed in tumor tissue compared to normal tissue in both the transcriptomic (UCSC Xena) and proteomic (CPTAC) datasets, suggesting its potential role in regulating aberrant proliferation in malignancies.

### 2.2. CASK Silencing Inhibits Keratinocyte Proliferation and Induces Cell Arrest at the G1 Phase

**Figure 2:**
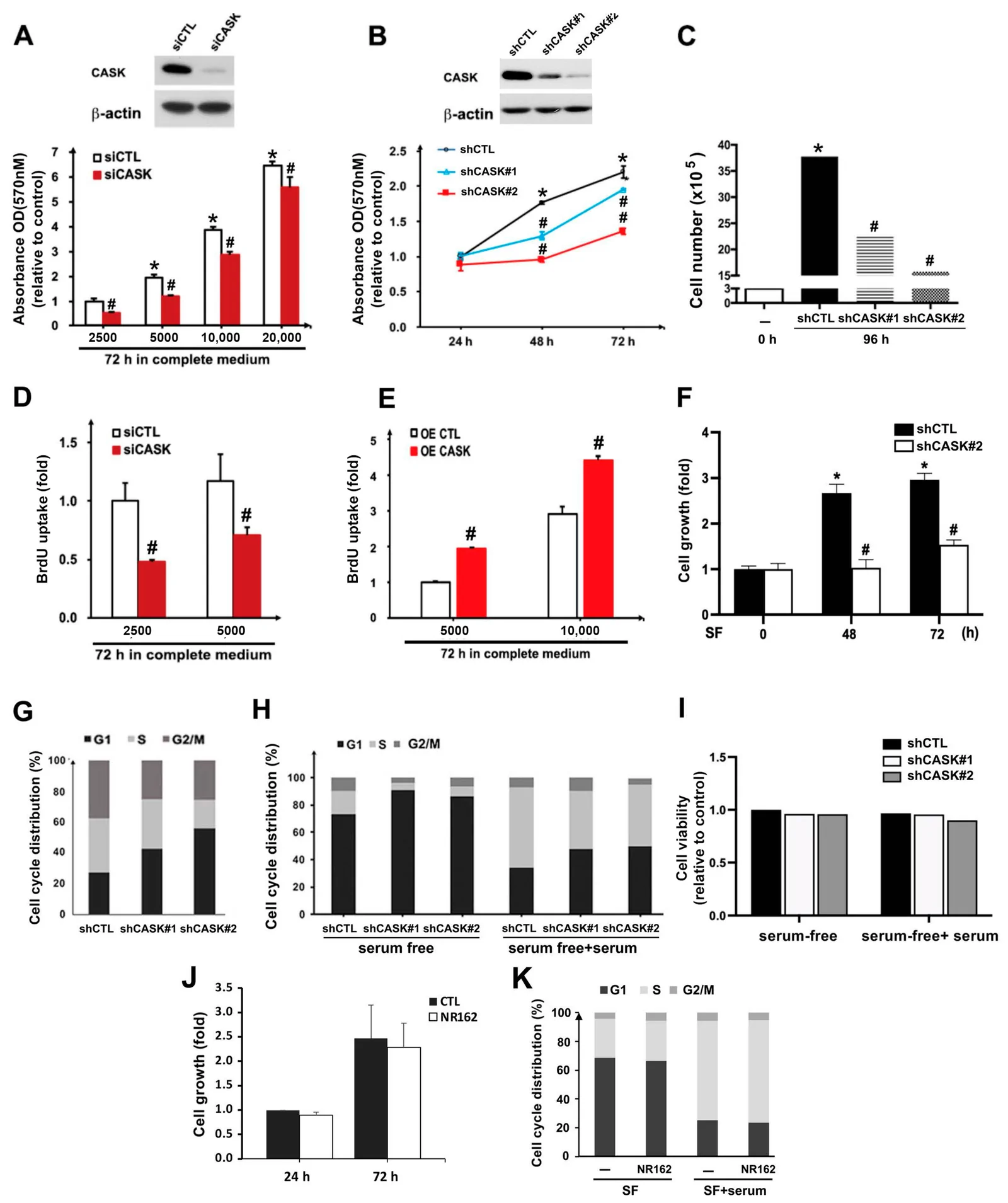
**CASK silencing reduces cell growth and cell cycle progression in HaCaT cells.** (**A**,**D**) HaCaT cells transfected with control siRNA or siCASK were cultured in complete medium at the indicated seeding densities for 72 h. (**B**,**C**) Stable shCTL and shCASK cells were serum-deprived and then cultured in complete medium for the indicated time. (**E**) Cells with CASK overexpression were established and cultured in complete medium for the indicated times. Cell proliferation was assessed by MTT assay (**A**,**B**), cell counting by trypan blue staining (**C**), and BrdU incorporation measured by chemiluminescence (**D**,**E**). (**F**) Cells cultured in complete medium were switched to serum-free medium, and viable cell numbers were determined at the indicated times using trypan blue exclusion. (**G**) Cells were cultured in complete medium for 48 h and stained with PI for cell cycle analysis. (**H**,**I**) Cells were cultured in serum-free medium for 24 h, followed by stimulation with or without 10% serum. After 16 h, the cell cycle was measured by PI staining and flow cytometry (**H**). After 72 h, cell viability was assessed by PI/Annexin V-FITC staining (**I**). (**J**,**K**) Control cells were cultured in complete medium, in the absence or presence of NR162 (2 μM), and similar experiments were conducted as in (**F**,**G**) to determine cell growth (**J**) and cell cycle analysis (**K**). The symbols (−) indicate the absence of the indicated treatment. * *p* < 0.05 indicates significant cell growth in control cells. # *p* < 0.05 indicates significant effects of CASK silencing or overexpression on cell growth compared with control conditions.

To understand the role of CASK, we silenced its protein expression in HaCaT cells by transfection with siRNA or shRNA. When cells were plated at different cell densities (2500–20,000 cells in 96-well plates) and grown for 72 h in a complete medium, the siCASK cell growth measured by MTT assay was significantly reduced (Figure 2A). The doubling time of HaCaT cells cultured in complete medium was around 48 h. Similarly, a reduction in viable cells was obtained in the shCASK group when cells were initially cultured in serum-free medium followed by complete medium for 3 days (Figure 2B). Using cell counting by trypan blue staining, we also observed a lower cell number in the shCASK group when 3 × 10^5^ HaCaT cells were plated and cultured in complete medium for 96 h (Figure 2C). Conducting a BrdU incorporation study in cells growing in complete medium, we found that siCASK led to a lower cell proliferation index (Figure 2D), while CASK overexpression increased BrdU incorporation (Figure 2E). Of note, HaCaT cells still can grow in the serum-free culture medium with a longer doubling time of 72 h, and this response was also blocked by CASK silencing (Figure 2F). All these data indicate that CASK is a positive regulator of keratinocyte proliferation. We further performed cell-cycle analysis using PI staining and flow cytometry after HaCaT cells were cultured in either a complete medium or a serum-free medium for 48 h. We found that loss of CASK expression resulted in a significant increase in the cell population in the G1 phase when continuously cultured in either a complete medium (Figure 2G) or a starvation condition (Figure 2H). After serum was added to pre-starved cells for 16 h, the increased cell population in the S phase was also attenuated in shCASK cells (Figure 2H). Next, we used Annexin V-FITC/PI staining to determine the role of CASK in cell apoptosis. We found that within 72 h of incubation in a complete medium or a serum-free medium, CASK silencing did not affect cell death (Figure 2I). These data further confirm that CASK can positively regulate keratinocyte proliferation without affecting cell death. To understand the role of kinase activity in the action of CASK, we treated cells with NR162 (a selective CaMK inhibitor of CASK). We found that NR162 did not alter cell growth (Figure 2J). Meanwhile, NR162 did not affect cell cycle progression (Figure 2K), indicating that CASK regulates keratinocyte proliferation in a kinase-independent manner.

### 2.3. CASK Silencing-Induced p21 Expression Contributes to Cell Cycle Arrest

**Figure 3:**
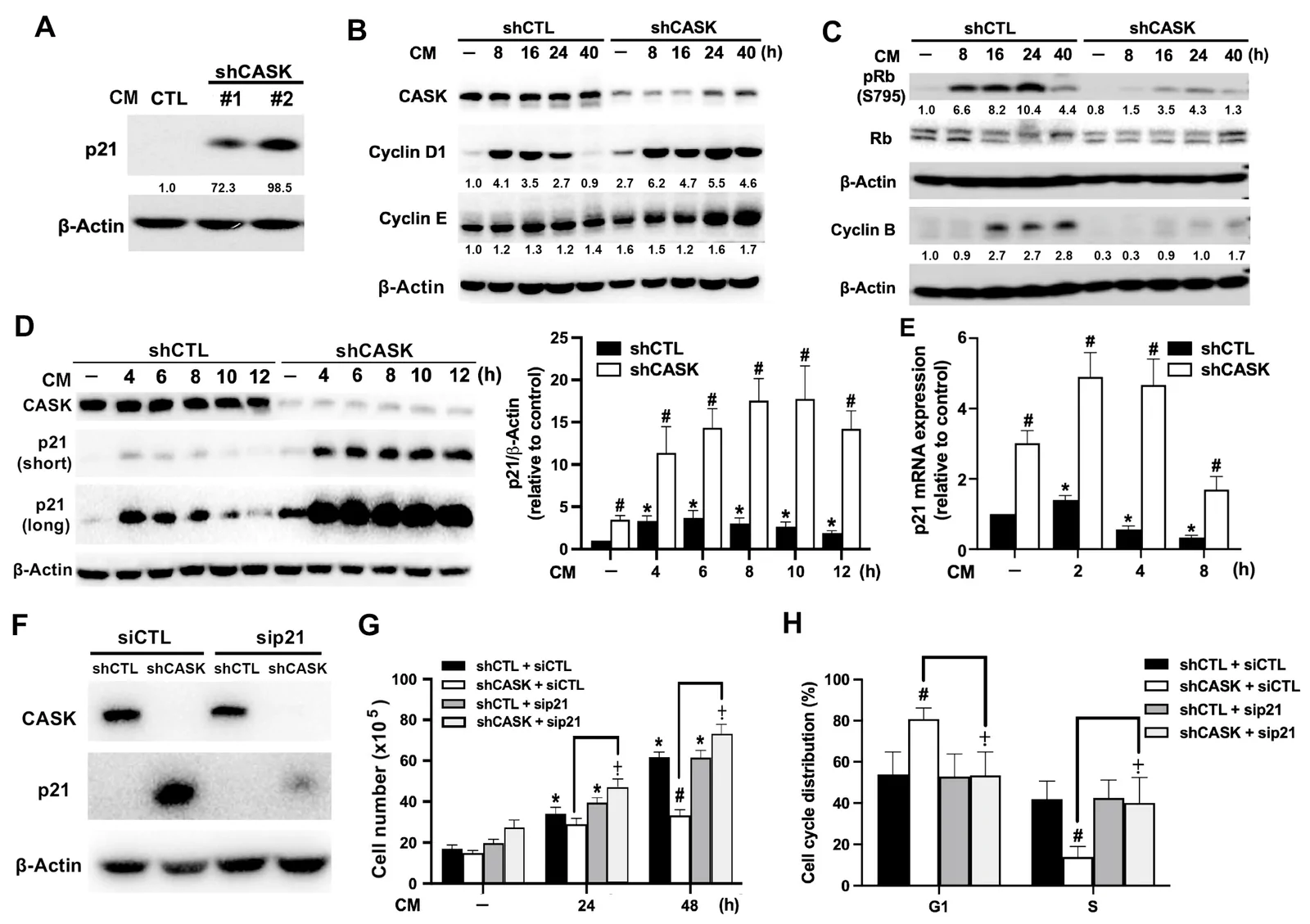
**CASK silencing promotes p21 expression that contributes to cell growth arrest.** (**A**) Control and shCASK cells were harvested, and protein levels of p21 were determined by immunoblotting. (**B**–**E**) Following 24 h of serum starvation, cells were maintained in either serum-free or complete medium for the indicated times. Cell lysates were then collected for immunoblotting analysis (**B**–**D**), and p21 mRNA expression was quantified by real-time PCR (**E**). (**F**–**H**) Control and shCASK cells were transfected with control siRNA or siRNA targeting p21 (sip21). Cells were maintained in serum-free medium for 24 h, followed by re-stimulation with complete medium for 24 h (**F**), the indicated times (**G**), or 6 h (**H**). Cell proliferation and cell cycle distribution were assessed by trypan blue exclusion (**G**) and PI staining (**H**), respectively. * *p* < 0.05 indicates significant effects of serum on p21 protein expression (**D**), gene expression (**E**), and cell growth (**G**). # *p* < 0.05 indicates significant effects of CASK silencing on the respective responses compared with control cells. + *p* < 0.05 indicates significant effects of p21 silencing in reversing shCASK-induced growth inhibition and cell cycle alterations.

After observing the effect of CASK silencing on increasing the cell cycle transition at the G1-S phase, we explored the regulatory molecules in this cell cycle stage. Previously, in human endothelial ECV304 cells, CASK has been shown to induce p21 protein and gene expression through inhibitor of differentiation 1 (Id1) and E2A signaling pathways, leading to inhibition of cell proliferation [31]. Here, we found that in complete medium culture, CASK silencing can upregulate p21 expression (Figure 3A). When serum was added to serum-deprived cells, the protein levels of cyclin D1 and cyclin E were increased, and this effect was enhanced in shCASK cells (Figure 3B). In contrast, the serum-induced cyclin B expression and Rb phosphorylation were attenuated in shCASK cells (Figure 3C). When starving cells were treated with 10% serum, the p21 protein level was increased at 4 h, then gradually declined within 12 h. We found that serum-induced p21 protein expression was markedly elevated and maintained in shCASK cells (Figure 3D). Accordingly, the mRNA level of p21 in shCTL cells was slightly and transiently increased at 2 h after serum treatment, followed by downregulation. In shCASK cells, increased p21 gene expression was observed in the resting state, and it was further enhanced after serum treatment (Figure 3E). Next, we investigated the role of p21 in CASK silencing-induced cell cycle arrest using a gene-silencing approach (Figure 3F). When measuring viable cells by trypan blue exclusion, we found that the growth inhibition observed in shCASK cells was reversed by p21 silencing (Figure 3G). Consistently, the effects of CASK silencing on cell cycle distribution, with increased cell number at the G1 phase and decreased cell number at the S phase, were similarly blocked in p21-silenced cells (Figure 3H). These findings indicate that CASK silencing can inhibit HaCaT cell growth by upregulating p21 gene expression.

### 2.4. CASK Silencing-Induced p21 Gene Expression Is Independent of p53 

**Figure 4:**
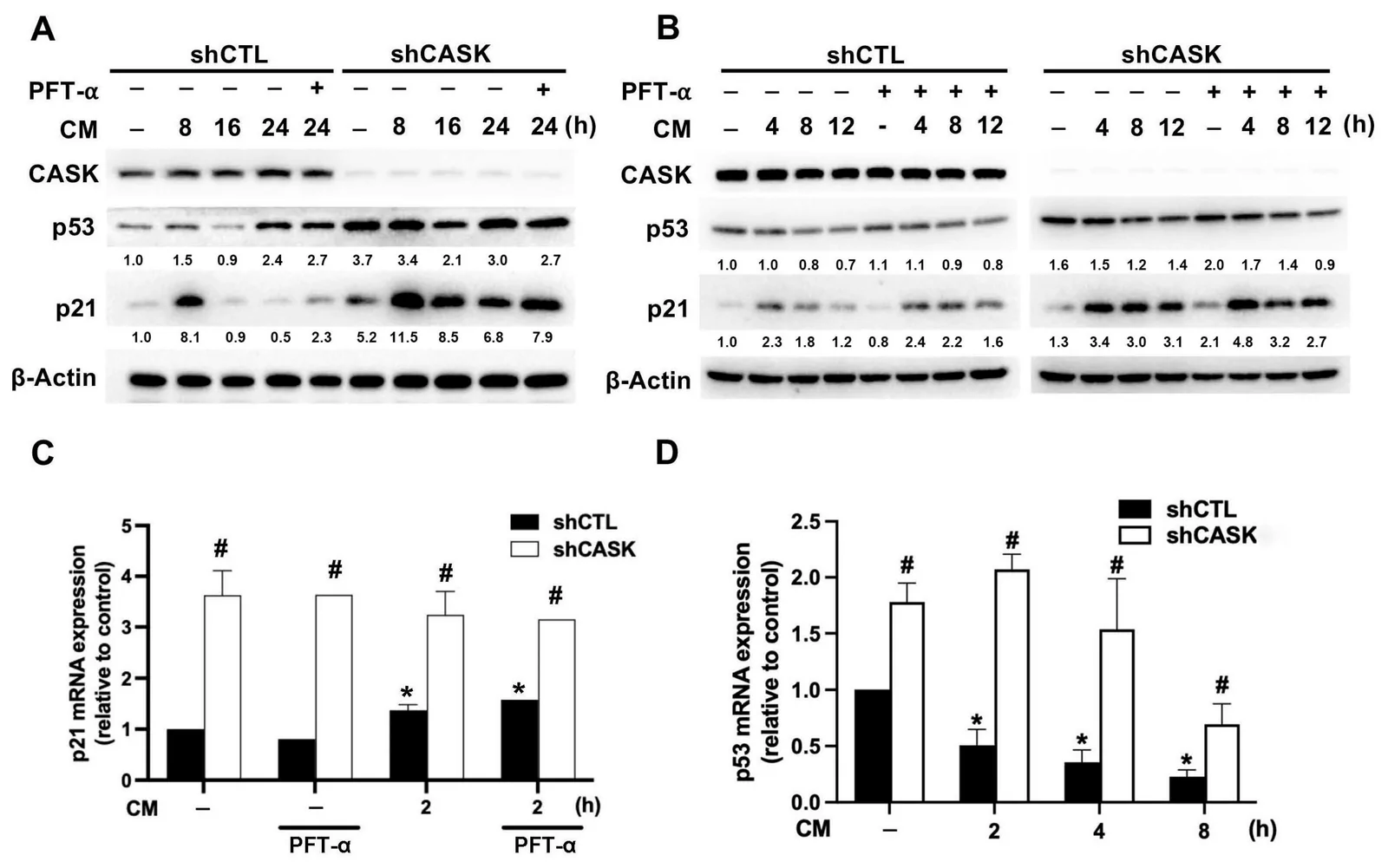
**CASK silencing promotes p21 protein expression.** Following 24 h of serum starvation, control and shCASK cells were transferred to complete medium and treated in the presence or absence of pifithrin-α (20 μM) for the indicated times. (**A**,**B**) Protein levels of p21 and p53 were analyzed by immunoblotting. (**C**,**D**) mRNA expression levels of p21 (**C**) and p53 (**D**) were quantified by real-time PCR. The (+) and (−) symbols indicate the presence and absence of the indicated treatment, respectively. * *p* < 0.05 indicates a significant effect of serum stimulation on gene expression. # *p* < 0.05 indicates a significant effect of CASK silencing on gene expression compared with control cells.

Because p21 is a target gene of p53, we wondered if p53 mediates shCASK-induced p21 gene expression. To this end, we measured p53 protein expression. We found that following the switch from serum-free to complete medium, the p53 protein levels gradually increased within 16–24 h (Figure 4A). Notably, shCASK cells exhibited elevated basal p53 protein levels, independent of serum presence or absence (Figure 4A,B). To investigate the p53–p21 axis, cells were treated with pifithrin-α, an inhibitor that modulates p53 post-translational modifications and suppresses p53-dependent transcription [32]. We found that the basal p21 protein level in serum-free conditions was increased by CASK silencing. Moreover, serum-induced short-term p21 protein expression was prolonged and elevated in shCASK cells, and this effect was not affected by pifithrin-α (Figure 4A,B). Consistent with previous reports, pifithrin-α also did not alter p53 protein levels [32]. Similarly, the p21 mRNA levels in serum-free and complete medium were higher in shCASK cells than in control cells, and this effect of CASK remained unchanged following pifithrin-α treatment (Figure 4C). To further explore the regulation of p53, we examined its mRNA expression. Serum stimulation led to a time-dependent reduction in p53 mRNA levels in control cells, whereas CASK silencing elevated p53 mRNA under serum-free resting conditions. Moreover, the serum-induced decline in p53 mRNA levels was markedly delayed in shCASK cells (Figure 4D). Collectively, these results suggest that p21 upregulation induced by CASK silencing occurs independently of p53.

### 2.5. CASK Silencing Increases EGFR Signaling Under Serum Re-Addition via Increasing EGFR Ligand Expression

**Figure 5:**
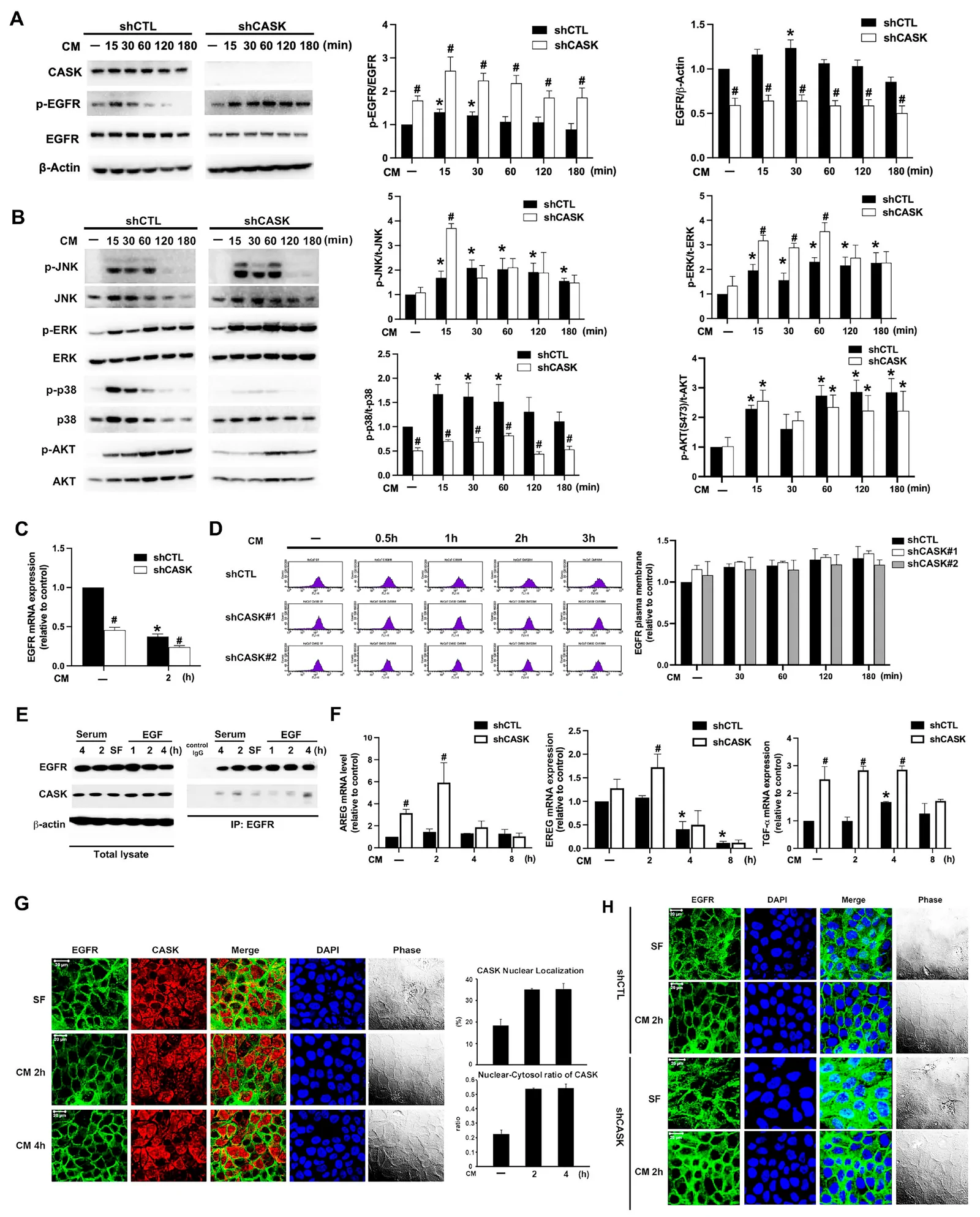
**CASK associates with EGFR and regulates serum-induced signaling and EGFR ligand expression.** After 24 h of starvation, control and shCASK cells were maintained in either serum-free or complete medium for the indicated times. (**A**,**B**) Cell lysates were collected and analyzed by immunoblotting. (**C**,**F**) mRNA expression levels of EGFR (**C**) and EGFR ligands (**F**) were quantified by real-time PCR. (**D**) Cell surface EGFR levels were assessed by antibody staining followed by flow cytometry. (**E**) Cell lysates were subjected to immunoprecipitation using EGFR antibodies, and co-immunoprecipitated CASK was detected by immunoblotting. * *p* < 0.05 indicates a significant response to serum stimulation. # *p* < 0.05 indicates a significant effect of CASK silencing on serum-induced responses. (**G**,**H**) After starvation for 24 h, HaCaT cells were either kept in serum-free or complete medium conditions for 2 and 4 h. HaCaT cells were stained with CASK and EGFR antibodies. Images were observed using confocal microscopy. In (**G**), CASK nuclear localization and the nuclear–cytosol ratio of CASK were quantified from immunofluorescence images. Data are presented as mean ± SEM.

EGFR activity plays a crucial role in keratinocyte proliferation and can shape p21 expression in keratinocytes induced by serum re-addition [25]. Keratinocytes, including HaCaT cells, have a constitutive EGFR signal due to high expression of EGFR ligands and EGFR [25,33], supporting the idea that EGFR activation under serum-free conditions primarily results from autocrine/paracrine mechanisms. Moreover, in complete medium, re-addition of serum introduces multiple EGF-family ligands (e.g., EGF, TGF-α, amphiregulin), which are present in serum and capable of activating EGFR. Therefore, we are interested in understanding if the regulation of cell growth in keratinocytes by CASK is related to EGFR activity. Another factor that drives this issue is that CASK has been shown to associate with EGFR in *C. elegans* and regulate EGFR function in basolateral localization [30]. First, we determined the effect of CASK silencing on serum-induced signaling. We found that CASK silencing can increase serum-induced EGFR, ERK, and JNK phosphorylation. However, serum-induced Akt phosphorylation was not affected by CASK silencing. In addition, the p38 phosphorylation and EGFR expression in both serum-free and complete medium conditions were attenuated in CASK-silencing cells (Figure 5A,B). Consistently, we observed a lower EGFR mRNA level in shCASK cells, and this effect is non-additive to that of serum re-addition (Figure 5C). This suggests the presence of a growth factor-dependent downregulation of EGFR gene transcription at the basal state of shCASK cells.

Next, we further determined the surface EGFR level and the interaction between CASK and EGFR. Using an EGFR antibody and flow cytometry, we did not observe a change in surface EGFR level after serum treatment in control and shCASK cells (Figure 5D). In addition, the data from the co-immunoprecipitation experiment showed the protein interaction between CASK and EGFR in different culture conditions, i.e., complete medium, serum deprivation, and EGF stimulation (Figure 5E). Given that HaCaT cells display a constitutively active autocrine/paracrine mode of EGFR signaling as mentioned above, we measured EGFR ligands. We found that the mRNA levels of EREG, but not those of AREG and TGF-α, were decreased by serum re-addition. In shCASK cells, these gene expressions were all increased under serum deprivation or serum re-addition (Figure 5F). These findings suggest that CASK silencing can induce autocrine/paracrine EGFR activation primarily via increasing EGFR ligand expression. To further understand if CASK can regulate EGFR distribution, we conducted confocal microscopy. Under serum-free conditions, EGFR was primarily localized at the plasma membrane, while CASK was distributed at the plasma membrane as well as in the cytosol and nuclei. In this condition, only minimal colocalization was observed between them. Upon serum stimulation, CASK underwent nuclear translocation, whereas EGFR remained predominantly at the plasma membrane (Figure 5G). Quantitative analysis revealed that the nuclear-to-cytoplasmic (N/C) ratio of CASK was increased from approximately 0.22 under serum-free conditions to 0.54 following 2 and 4 h of serum stimulation, indicating increased nuclear accumulation of CASK upon serum stimulation. Notably, CASK silencing did not significantly affect EGFR localization, either in the serum-free or in the complete medium conditions (Figure 5H). All these findings indicate that CASK silencing can increase EGFR activation regardless of the presence or absence of serum, and this effect might partially result from the increased expression of EGFR ligands. 

### 2.6. The Autocrine EGFR-ERK Axis Contributes to Serum-Induced p21 Expression Under CASK Silencing

**Figure 6:**
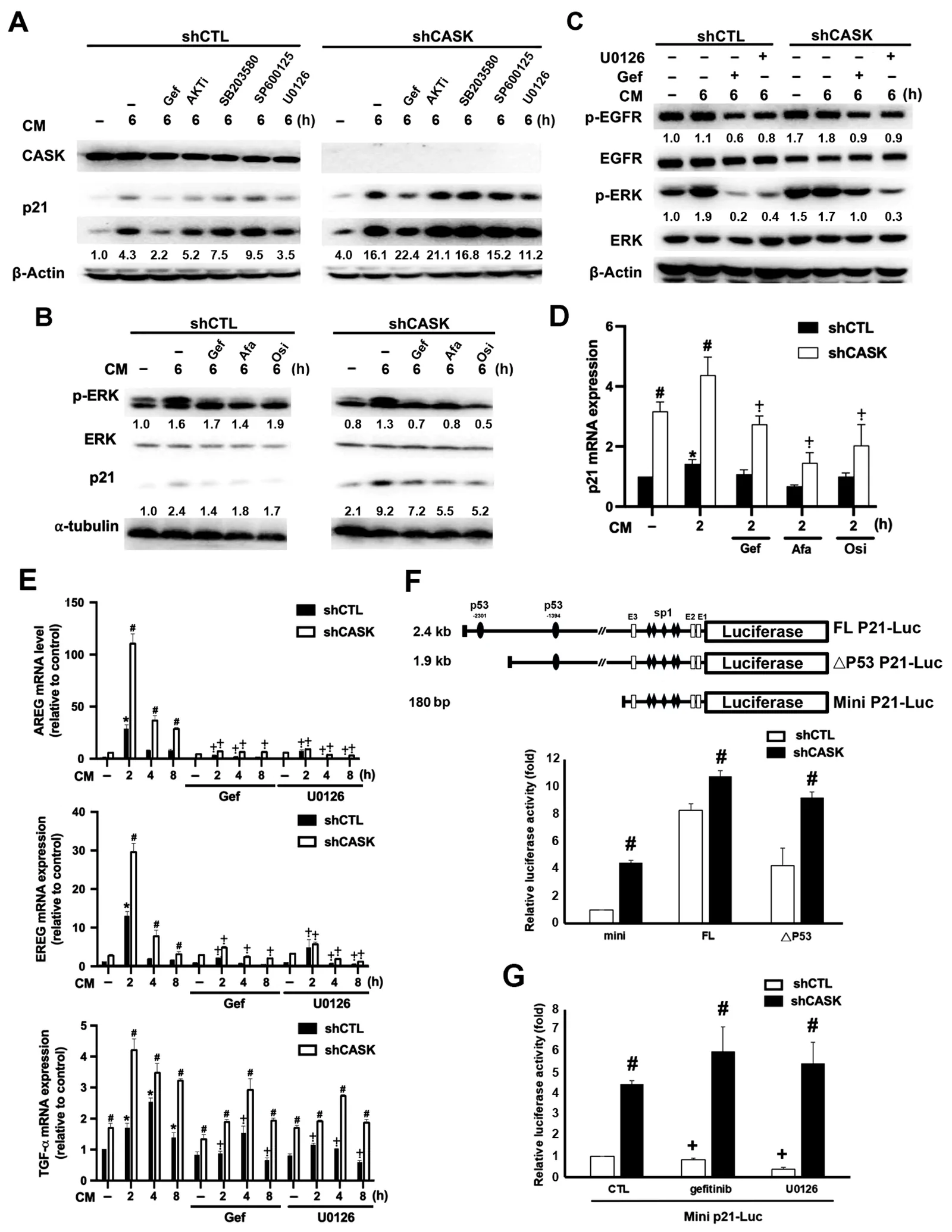
**EGFR TKIs and ERK inhibition suppress serum-induced expression of p21 and EGFR ligands, and p21 promoter activity.** After 24 h of serum starvation, control and CASK-silenced cells were stimulated with serum for the indicated times, in the absence or presence of gefitinib (2.5 µM), afatinib (0.5 µM), osimertinib (1 µM), AKTi (10 µM), SB203580 (10 µM), SP600125 (10 µM), or U0126 (10 µM). (**A**–**C**) Cell lysates were collected and analyzed by immunoblotting. (**D**,**E**) The mRNA expression levels of p21, AREG, EREG, and TGF-α were quantified by real-time PCR. (**F**) Control and shCASK cells cultured in complete medium were transfected with p21 luciferase reporter plasmids as indicated for 16 h, and luciferase activity reflecting p21 promoter activation was measured using a luminescence-based assay. (**G**) Cells transfected with the p21 luciferase mini-reporter plasmid (spanning the −180 to +8 bp region) were treated with gefitinib (2.5 μM) or U0126 (10 μM) for 16 h, followed by measurement of luciferase activity. * *p* < 0.05 indicates a significant response to serum stimulation. # *p* < 0.05 indicates a significant effect of CASK silencing on serum-induced responses. + *p* < 0.05 indicates a significant effect of EGFR TKIs and U0126 on shCASK-induced p21 expression.

To date, most studies reveal that the strong p21 induction by serum re-addition [30], mitogenic stimuli [24,25,34], and oxidative stress [35] is independent of p53. Moreover, EGFR activation contributes to p21 expression in many cell types [21,25,36], including in keratinocytes [24]. Mechanistically, EGFR-ERK is involved in EGFR-dependent p21 expression [23]. After observing higher EGFR activation in shCASK cells, we used pharmacological agents to decipher the signaling mechanism for p21 regulation. These include EGFR TKIs (gefitinib, afatinib, osimertinib), ERK inhibitor (U0126), Akt inhibitor AKTi, JNK inhibitor (SP600125), and p38 inhibitor (SB203580). We found that p21 protein expression induced by serum in both shCTL and shCASK cells was inhibited by gefitinib, afatinib, osimertinib, and U0126, but not by others (Figure 6A,B). Moreover, ERK activation by serum was inhibited by EGFR TKIs (Figure 6B,C). The real-time PCR data also showed that the serum-induced mRNA expression of p21 (Figure 6D) and EGFR ligands (e.g., AREG and EREG) (Figure 6E, upper and middle panels) in both shCTL and shCASK cells was abolished by gefitinib and U0126. On the other hand, serum-induced TGF-α gene expression was moderately inhibited by gefitinib and U0126 in shCTL and shCASK cells, while both inhibitors did not significantly change the enhancing effect of CASK silencing (Figure 6E, lower panel). We suggest the existence of autocrine/paracrine action and a positive feedback loop of EGFR activation in keratinocytes cultured in complete medium conditions. Moreover, such constitutive EGFR activation contributes to ERK-dependent gene expression of EGFR ligands and p21.

To further elucidate the molecular mechanisms of p21 gene expression controlled by CASK, we conducted a reporter assay using the full-length (FL) wild-type promoter and a version lacking the p53-responsive elements. Consistent with our data on p21 mRNA expression, CASK silencing significantly increased p21 promoter activity. Although deletion of the p53-responsive elements reduced luciferase activity in control cells, CASK-silenced cells still displayed its enhancement effect (Figure 6F), suggesting that p21 gene expression in the resting state might be dependent on p53, while upregulation of p21 expression by CASK silencing might be independent of p53. We next tested a minimal 180 bp p21 promoter construct, which harbors the −180 to +8 base pair region of the p21 promoter. Remarkably, CASK knockdown again led to a pronounced increase in luciferase activity (Figure 6F). Although treatment with gefitinib or U0126 significantly reduced the reporter activity of the minimal p21 promoter in the resting state, CASK silencing still exhibited its enhancing effect (Figure 6G). These findings reveal that the region between –180 bp and the TATA box of the p21 promoter is the key regulatory site targeted by CASK.

## 3. Discussion

CASK is a ubiquitously expressed multifunctional scaffold protein whose diverse biological functions are largely attributed to its ability to interact with a wide range of protein partners. The role of CASK in regulating cell growth appears to be highly context- and cell type-dependent. Several studies have demonstrated that CASK promotes cell proliferation and tumor progression in multiple cancer types [7,37,38,39]. In contrast, earlier studies reported a growth-suppressive function of CASK in epidermal keratinocytes [11] and endothelial ECV304 cells [40]. Mechanistically, CASK was shown to inhibit cell cycle progression through enhancement of p21 promoter activity via interaction with Id1 and by facilitating E2A-dependent p21 transcription in ECV304 cells [40]. Interestingly, our findings differ from the previous observations in HaCaT keratinocytes [11]. In the present study, we identified CASK as a positive regulator of keratinocyte proliferation. Importantly, this pro-growth effect was independent of its CaM kinase activity and was instead associated with attenuation of EGFR-ERK signaling-mediated p21 expression. These findings suggest that CASK may exert distinct regulatory effects on cell proliferation depending on the cellular context and signaling environment, underscoring the complexity of its biological functions in epithelial cells.

EGFR signaling plays a critical role in maintaining the balance between keratinocyte proliferation and differentiation. Sustained EGFR activity promotes keratinocyte proliferation, whereas modulation of this pathway can shift cellular programs toward differentiation [19,20]. As a key regulator of cell cycle progression, p21 serves as a central mediator of keratinocyte growth control [21]. Importantly, p21 functions as a signaling hub that integrates inputs from multiple regulatory pathways, including growth factor-mediated signaling. Previous studies have demonstrated that ERK activation induces p21 expression in HaCaT cells at both the mRNA and protein levels [23,27,28]. Because serum contains ligands for the EGFR family, serum re-stimulation activates EGFR and its downstream ERK cascade, resulting in p21 upregulation in keratinocytes [23] as well as other epithelial cells [24]. Furthermore, the EGFR-ERK-p21 regulatory axis has been documented in a variety of cell types, including fibroblasts [25], A431 epidermoid carcinoma cells [26], squamous cell carcinoma cells [41], enterocytes [42], head and neck cancer cells [43], and glioblastoma cells [36], suggesting that this pathway represents a broadly conserved mechanism for controlling cell proliferation. Consistent with these previous findings, our results confirm the existence of the EGFR-ERK-p21 signaling axis in keratinocytes and further identify CASK as a previously unrecognized negative regulator of this pathway. Several lines of evidence support this notion. First, CASK silencing enhances both EGFR and ERK activities. Second, pharmacological inhibition of EGFR with EGFR TKIs or inhibition of ERK activation with U0126 effectively suppresses p21 gene expression in shCASK cells. In contrast, other EGFR-associated signaling pathways, including AKT, p38 MAPK, and JNK, appear to be dispensable for p21 regulation, as selective inhibitors of these kinases fail to alter p21 expression. Collectively, these findings indicate that the EGFR–ERK axis, rather than other canonical EGFR downstream pathways, is primarily responsible for mediating p21 upregulation in shCASK cells. Notably, a similar growth-promoting role of CASK was observed in our recent study of non-small cell lung cancer, in which CASK enhanced cancer cell proliferation and tumor progression [39]. Collectively, these findings support a model in which CASK facilitates cell growth, at least in part, through attenuation of the EGFR-ERK-p21 growth-inhibitory signaling axis.

The p21 promoter contains two major p53-responsive elements, located at approximately −2301 and −1394, which mediate transcriptional activation of p21 in response to DNA damage and various cellular stresses [34]. In the present study, serum re-addition following starvation resulted in a modest reduction in p53 mRNA and protein expression during the early phase, while simultaneously inducing a transient increase in p21 expression at both the mRNA and protein levels. The serum-mediated suppression of p53 may be attributable to growth factor-dependent activation of the PI3K/AKT pathway, which has been reported to repress p53 transcription and promote destabilization of both p53 mRNA and protein [44,45,46]. In parallel, sustained ERK activation induced by serum re-stimulation has been shown to promote p21 expression through a p53-independent mechanism, thereby contributing to cell cycle arrest and growth control [25,34,47,48]. Within this context, we found that CASK silencing significantly increased p53 mRNA and protein expression in keratinocytes cultured under serum-free conditions. Although the underlying mechanism by which CASK deficiency enhances p53 expression remains unclear, our data suggest that p53 might not be required for the induction of p21 following CASK knockdown. This conclusion is supported by several lines of evidence. First, pharmacological inhibition of p53 using PFT-α failed to attenuate CASK silencing-induced p21 mRNA and protein expression. Second, deletion of the p53-responsive elements from the p21 promoter did not alter the ability of CASK knockdown to enhance p21 promoter activity. Together, these findings indicate that the regulation of p21 by CASK is more likely mediated by alternative signaling pathways, such as the EGFR-ERK axis identified in the present study.

However, despite the above suggestions on p53, one unique issue regarding p53 status in HaCaT cells still requires clarification. The p53 protein in HaCaT cells harbors gain-of-function (GOF) mutations (H179Y and R282Q) [49], making the regulation of p21 expression in these cells particularly complex. Due to conformational alterations, mutant p53 is unable to efficiently bind the p53-responsive element within the p21 promoter and therefore cannot directly transactivate p21 expression in HaCaT cells [50]. Instead, mutant p53 can interact with p63, a member of the p53 family that functions as a transcriptional repressor of p21, thereby preventing p63 from binding to the p21 promoter and relieving its suppressive effect on p21 expression [51]. In addition, mutant p53 has been shown to redirect p63 to alternative genomic binding sites, resulting in extensive reprogramming of the p63 transcriptional network [52,53]. Consequently, mutant p53 may still regulate p21 expression indirectly through p63-dependent mechanisms. Given the unique p53 status of HaCaT cells, it remains important to determine whether the p53-mutant/p63 axis contributes to p21 upregulation following CASK silencing. Further investigation will be required to clarify the involvement of this pathway and its potential interaction with the EGFR–ERK signaling cascade in regulating p21 expression under CASK-deficient conditions.

Our data suggest that transcription factors that bind to the p21 promoter within −180 bp possibly mediate the function of CASK. Given that CASK-associated regulation of p21 expression is dependent on ERK, several ERK-responsive transcription factors that bind to the p21 promoter within −180 bp are speculated. These are Sp1 [54,55], E2A [56,57], and c-Jun [58]. Sp1 binds to the GC-rich proximal promoter region (−82/−77) and is critical for p21 transcription via p53-dependent and -independent mechanisms [59,60,61]. Binding of E2A to E1- and E2-boxes at −21 to +1 bp and to the E3-box within −162 to −157 bp drives p21 transcription [56]. All these transcription factors have been shown to mediate serum-induced p21 transcription. As mentioned above, CASK has been shown to upregulate p21 via the E2A transcription factor in ECV304 cells, via binding to Id1, impairing interaction between Id1 and E2A, and allowing free E2A binding to the p21 promoter [31]. In the future, we still need to decipher the molecular mechanisms of the major transcription factors contributing to CASK-dependent p21 expression in keratinocytes.

Keratinocytes, including HaCaT cells, exhibit constitutively active EGFR signaling owing to the abundant expression of both EGFR and its ligands, as well as continuous secretion of EGFR ligands such as HB-EGF, amphiregulin, and TGF-α [19,33,62,63,64]. The expression of these ligands is largely regulated by the RAS-RAF-MEK-ERK signaling cascade [65,66,67], thereby establishing a sustained autocrine/paracrine positive-feedback loop that maintains EGFR activity even under serum-free conditions. This regulatory circuit is essential for supporting keratinocyte proliferation, survival, and wound-healing responses [19,62,68]. Notably, EGFR activation, whether triggered by serum stimulation or autocrine ligand production, has been reported to suppress EGFR gene expression while preserving ligand-mediated signaling activity [19,69]. Consistent with this notion, we also observed lower EGFR gene expression alongside increased EGFR ligand expression and enhanced EGFR phosphorylation in shCASK cells and following serum re-addition. Nevertheless, our previous study demonstrated that CASK silencing promotes EGFR mRNA and protein levels in microglial cells [70] and non-small cell lung cancer [39]. Collectively, these results suggest that CASK functions as an important modulator of EGFR signaling by coordinately regulating the expression of both EGFR and its ligands, thereby fine-tuning autocrine/paracrine EGFR activity. The distinct effects of CASK on EGFR signaling observed across different cell types further indicate that the underlying regulatory mechanisms are highly context-dependent and may vary according to cellular lineage, signaling networks, and microenvironmental cues. Further investigation is therefore required to elucidate the molecular basis of CASK-mediated regulation of EGFR signaling in different biological settings.

## 4. Conclusions

CASK is a multifunctional scaffold protein involved in the regulation of cell proliferation. In keratinocytes, CASK suppresses autocrine/paracrine EGFR signaling, thereby limiting constitutive ERK activation. Through this mechanism, CASK inhibits p21 expression and increases keratinocyte proliferation, highlighting its role as a negative regulator of the EGFR-ERK-p21 signaling axis.

## 5. Materials and Methods

### 5.1. Reagents and Antibodies

Dulbecco’s Modified Eagle Medium, trypsin/EDTA solution, and fetal bovine serum (FBS) were purchased from Gibco (Carlsbad, CA, USA). Penicillin–streptomycin (P/S) solution was purchased from Biological Industries (Kibbutz Beit Haemek, Israel). PBS, sodium bicarbonate, puromycin, and 3-(4,5-dimethylthiazol-2-yl)-2,5-diphenyltetrazolium bromide (MTT) were bought from Sigma-Aldrich (St. Louis, MO, USA). ERK inhibitor U0126 was bought from Calbiochem (San Diego, CA, USA). Gefitinib, afatinib, and osimertinib were purchased from Selleckchem (Houston, TX, USA). NR162, pifithrin-α, SP600125, SB203580, and AKT-IN-10 (AKTi) were bought from MedChemExpress (Monmouth Junction, NJ, USA). Antibodies of p-EGFR (Y1068) (#3777S), p-JNK (Thr183/185) (#9251), p-ERK (Thr202/Tyr204) (#9101), p-p38 (Thr180/Tyr182) (#9211), p-AKT (Ser473) (#4060S), AKT (#9272S), p53 (#9282), pRb (S795) (#9301), and Rb (D20) (#9313) were purchased from Cell Signaling Technology (Beverly, MA, USA). Antibodies of CASK (sc-13158), β-actin (sc-47778), JNK1 (sc-1648), p38α/β (sc-7972), ERK1/2 (sc-514302), cyclin B (sc-166210), cyclin D1 (sc-718), cyclin E (sc-25303), and p21 (sc-53870) were purchased from Santa Cruz Biotechnology (Dallas, TX, USA). The EGFR antibody (06-847) was purchased from Merck Millipore (Burlington, MA, USA).

### 5.2. Cell Lines and Cell Culture

Human immortalized keratinocytes, HaCaT cells, were cultured in high-glucose DMEM medium supplemented with 10% FBS and 1% penicillin–streptomycin-amphotericin B. Cells were incubated at 37 °C under a humidified atmosphere of 5% CO_2_.

### 5.3. CASK Silencing Using siRNA and shRNA Approaches

For siRNA, cells were transfected with either pooled control non-specific siRNA (100 nM) or pooled siRNA of human CASK (100 nM) in OPTI-MEM with 10% FBS. The transfection was performed using DharmaFECT Transfection Reagents (Thermo Fisher Scientific, Lafayette, CO, USA) according to the manufacturer’s protocol. Twenty-four hours after transfection, cells were trypsinized and seeded in cell culture plates overnight before treatment with the indicated stimuli. In the construction of shRNA knockdown cell lines, lentiviral particles encoding shRNA targeting CASK were used for transfection. Lentivirus-containing supernatants were harvested 24 h after transfection, filtered using a 0.45 μm filter, and diluted with fresh culture media to transduce target cells in the presence of 8 µg/mL PolyBrene (hexadimethrine bromide) (Monmouth Junction, NJ, USA). Transduced cells were treated with puromycin (3 μg/mL) for 2 weeks to select for successful transfection. The silencing effect was evaluated by immunoblotting.

### 5.4. Silencing p21 in Control and shCASK Cells

In some experiments, p21 gene expression in control and shCASK cells was silenced by transfection with either pooled control non-specific siRNA (100 nM) or pooled siRNA of p21 in OPTI-MEM with 10% FBS. The transfection was performed using DharmaFECT Transfection Reagents (Thermo Fisher Scientific, Lafayette, CO, USA). Twenty-four hours after transfection, cells were trypsinized and seeded in cell culture plates overnight before treatment with the indicated stimuli. 

### 5.5. Immunoblotting Analysis

Cells were seeded in 6-well plates at a density of 5 × 10^5^ and cultured in the medium either with or without serum for 1 day before treatment with the indicated agents. After treatment for the indicated periods, the medium was removed, and the cells were washed with PBS twice. Subsequently, the cells were lysed with 1× sample loading buffer and underwent sonication with 10 interval pulses, then heating at 95 °C for 10 min. Protein sample lysates were loaded and separated on a 6–15% SDS-PAGE gel, and then further electro-transferred onto an ImmunoBlot P transfer membrane (Millipore, Bedford, MA, USA). The membrane was blocked and shaken gently with 5% non-fat milk in Tris-buffered saline (TBS) with 0.1% Tween 20 for 1 h at room temperature. The membrane was washed 3 times with an interval of 10 min before the primary antibody incubation at 4 °C. After overnight incubation with the primary antibody (1:1000 dilution), the membrane was washed three times with TBST at 10-min intervals and then incubated with an HRP-conjugated secondary antibody (1:5000 dilution) for 1 h at room temperature. The membrane was subsequently washed three times with TBST for a total of 1 h before detection. The membrane was soaked in the chemiluminescence reagents, and the protein bands on the membrane were determined using the Bio-Rad UVP system (Bio-Rad, Hercules, CA, USA). The protein blots were quantified using Imagelab (version 6.1) analysis, and β-Actin was used as the internal control to indicate an equal amount of protein loading.

### 5.6. RNA Extraction, Reverse Transcription, and Real-Time PCR (Q-PCR)

After stimulation and treatment, cells were washed with PBS and lysed with 300 μL TRIzol reagent (Invitrogen, Shanghai, China) for RNA extraction. RNA was extracted according to the manufacturer’s protocol; 2 μg of RNA was converted to cDNA using the reverse transcription system kit (Promega, Heidelberg, Germany). For qPCR determination, FastStart SYBR Green Master (Roche Diagnostics, Indianapolis, IN, USA), targeted gene primers (Supplementary Table S1), and ddH_2_O were mixed with cDNA in 96-well plates and analyzed using ABI Prism 7900 (Applied Biosystems, Oakland, CA, USA). The level of mRNA change was analyzed by normalizing to the housekeeping gene β-actin.

### 5.7. Co-Immunoprecipitation

Cells were seeded in 6 cm culture dishes and lysed with 500 μL lysis buffer (50 mM Tris-HCl, pH 7.6, 150 mM NaCl, 1% Triton X-100, 2 mM EDTA, 2 mM NaF, 2 mM NaVO_3_, 1 mM phenylmethylsulfonyl fluoride, and 0.2% protease inhibitor cocktail). After sonication and centrifugation at 12,000 rpm for 25 min at 4 °C, supernatants were collected. For the pre-clearing step to reduce non-specific binding to the protein A/G agarose beads, 1 μL normal IgG antibody and 10 μL protein A/G beads were added to each sample and rocked gently at 4 °C for 30 min. After spinning down the beads and transferring the supernatants to a fresh tube, 1 μg of antibody against target proteins or control normal IgG antibody was added to the tubes. The samples were incubated in a 4 °C cold room with gentle rocking overnight. Protein A/G beads (10 μL) were added and incubated in a 4 °C cold room with gentle rocking for 1 h. The sample was washed twice with lysis buffer, followed by once with lysis buffer at a higher concentration of NaCl (300 mM), and twice again with lysis buffer. Resuspend the pellets with 50 μL 1× sample loading buffer and heat at 95 °C for 5 min. Samples were analyzed by Western blotting.

### 5.8. Cell Growth Determination by MTT Assay and Trypan Blue Staining

The cells were seeded in 48-well or 96-well plates in 100 μL medium and treated with the indicated agents. Then, 1 mg/mL MTT (Thiazolyl Blue Tetrazolium Bromide; Sigma-Aldrich, St. Louis, MO, USA) was incubated for 3 h. MTT produces a yellowish solution that is converted to dark blue, water-insoluble MTT formazan crystals by mitochondrial dehydrogenases of living cells. The whole medium with the MTT solution was then removed and replaced with 100 μL DMSO to dissolve the purple crystals. The plate was put on a shaker for 5 min to allow the blue crystals to dissolve evenly. Finally, the intensity was measured colorimetrically at 550 nm and 630 nm and calculated as the relative viability of cells. For trypan blue staining experiments, cells (1 × 10^5^/well) were seeded in a 6-well plate and incubated in culture medium either with or without serum. Cells were trypsinized from the 6-well plate at the indicated time point and centrifuged at 1500 rpm for 6 min. The cell pellets were collected and resuspended in fresh medium. Then, 10 μL of cell suspension was mixed well with 10 ul of trypan blue dye, and the mixture of cell suspension and trypan blue was loaded into the hemocytometer. Live cells (clear) are counted, while dead cells (blue) are excluded while conducting the cell count studies.

### 5.9. BrdU Uptake 

After overnight serum starvation, cells were incubated for the indicated times, and cell proliferation was assessed using a BrdU ELISA kit (Roche, Cat. No. 11669915001, Indianapolis, IN, USA) according to the manufacturer’s instructions. Briefly, 10 μL of BrdU labeling solution was added to each well for 4 h, and BrdU incorporation was detected using a BioTek Synergy H1 microplate reader (Agilent Technologies, Santa Clara, CA, USA).

### 5.10. Cell Cycle Analysis

Cells (2 × 10^5^ per well) were seeded in 6-well plates in serum-free DMEM medium. After overnight attachment, the medium was replaced with complete medium for the indicated durations. Cells were then harvested by trypsinization, collected by centrifugation at 1000 rpm for 5 min, and fixed in 75% pre-chilled ethanol at −20 °C overnight. Following fixation, cells were pelleted, washed twice with PBS, and resuspended in 500 μL PI/RNase staining buffer (Cat. No. 550825). After incubation for 30 min at room temperature in the dark, cell cycle distribution was analyzed by flow cytometry using a FACSCalibur system (BD Biosciences, Franklin Lakes, NJ, USA), and the data were processed with ModFit LT™ software (version 5.1).

### 5.11. Annexin V/PI Staining

After the indicated treatment, the culture medium (including detached dead cells) was collected. The remaining adherent cells were trypsinized and pooled with the previously collected medium in the same Eppendorf tube. After centrifugation, the supernatant was removed, and the cell pellets were stained with 100 μL Annexin V binding buffer containing 5 μL of Annexin V and 10 μL of propidium iodide (PI) for 20 min at room temperature. After incubation, the stained cells were transferred into a flow tube, and 400 μL Annexin V binding buffer was added. Cell viability was measured by flow cytometry (FACSCalibur, BD, Franklin Lakes, NJ, USA) with the FL1 and FL2 bivariate analysis. The results were next analyzed with the CellQuest Pro software 4.0.1 by calculating the cell percentage in the lower left quadrants as viable cells and the remaining quadrants as dead cells. 

### 5.12. Immunofluorescence 

After transfection of CASK silencing and silencing control, cells were treated with full serum or serum-free medium for 24 h. EGF was added to starving cells for the indicated periods. Then, the cells were fixed with 4% paraformaldehyde in PBS for 20 min. After washing with PBS three times, the cells were permeabilized with 0.2% Triton X-100 in PBS for 20 min. After fixation, the samples were blocked with 5% BSA for 1 h and then incubated with primary antibodies against EGFR and CASK at 1:300 and 1:200 dilutions for 2 h at room temperature or overnight at 4 °C after aspiration of the blocking solution. The primary antibody was then discarded, and cells were washed three times with PBS. The samples were incubated with fluorochrome-conjugated secondary antibody (1:2000 dilution) for 1 h in the dark afterwards. Following the immunostaining process, the coverslip was counterstained with 4′,6-diamidino-2-phenylindole (DAPI) and mounted on microscope slides in the dark. Samples were analyzed by an LSM 780 confocal microscope (Zeiss, Oberkochen, Germany).

### 5.13. Flow Cytometry for Surface EGFR Expression

Cells (1 × 10^5^/well) were seeded in a 6-well plate and incubated in culture medium either with or without serum. EGF (50 ng/mL) was added to the serum-starved cells for the indicated time points. Trypsin was used to collect the cells, and the cells were centrifuged at 1100 rpm for 5 min in 1.5 mL microtubes. The medium was aspirated, and the cells were incubated in 0.5% BSA in PBS for 30 min on ice. The cells were centrifuged at 1100 rpm for 5 min, and the blocking solution was removed. Then, 0.8 μg/mL Alexa Fluor^®^ 488 anti-human EGFR Antibody (#352908, BioLegend, San Diego, CA, USA) was added to the 0.5% BSA solution in each reaction. Anti-mouse IgG F(ab’)2 Alexa Fluor^®^ 488 Conjugate (#4408, Cell Signaling, Beverly, MA, USA) was used as a negative control. After incubating on ice for 30 min, the cells were centrifuged at 1100 rpm for 5 min and resuspended in 500 μL PBS before being transferred into flow tubes. The fluorescence intensity of surface EGFR was detected by flow cytometry with the FACSCalibur system (BD Bioscience, San Jose, CA, USA) and analyzed by FlowJo software (version V10).

### 5.14. p21 Promoter Luciferase Assay

Cells were co-transfected with the p21 reporter plasmid and the β-galactosidase expression vector (pCMV-β-Gal) using Lipofectamine 2000 Transfection reagent (Invitrogen, Shanghai, China). After treatment with the indicated reagents, the cells were lysed in a reporter lysis buffer (Promega, Madison, WI, USA), and the lysates were reacted with a commercial luciferase substrate provided in the luciferase assay system kit (Promega) and assayed on a microplate luminometer (Packard, Meriden, CT, USA). Luciferase activity values were normalized to transfection efficiency monitored by β-galactosidase expression and presented as the percentage of luciferase activity of the control group. The p21 reporter plasmids containing wt (−2400 to +8), minimal (−180 to +8), and p53 binding site deleted p21 promoter construct (−1866 to +8) were gifts from B. Vogelstein (Johns Hopkins University, Baltimore, MD, USA).

### 5.15. RNA-Seq Analysis

Following quality control and concentration assessment, differential expression (DE) analysis was conducted using the LPEseq algorithm. Significant DE genes (DEGs) were defined by a fold change threshold of >1.5 and a false discovery rate (FDR) < 0.01. To dissect the role of the CASK gene in mediating cellular adaptation to metabolic stress, four pairwise DE comparisons were performed across two variables, namely genotype (shCTL vs. shCASK) and environmental condition (CM vs. SF). To integrate DE magnitude and statistical significance, the π-score was calculated for each gene as the product of log2(FC) and −log10(FDR). To specifically quantify the degree to which CASK loss modifies the serum starvation response, an interaction score (ΔΔπ) was computed as follows: ∆∆π = π (shCTL_CM vs. shCTL_SF) − π (shCASK_CM vs. shCASK_SF). Genes ranked by π or ΔΔπ scores were subjected to Gene Set Enrichment Analysis (GSEA) using the GSEApy package (v1.1.11). The raw and processed RNA sequencing data generated in this study have been deposited in the NCBI Gene Expression Omnibus (GEO) under accession number GSE336963.

### 5.16. Pathway Enrichment Network and Clustering

A pathway enrichment network was constructed by calculating Jaccard similarity scores based on shared lead genes between pathways. The Leiden clustering algorithm was subsequently applied to identify distinct functional modules within the network. These clusters were semantically annotated with representative biological terms. To identify key regulatory drivers in each functional module, “core lead genes” were defined as those possessing a degree centrality > 0.8 relative to the total number of pathways within their respective cluster.

### 5.17. Clinical Validation with an External Database

To evaluate the clinical relevance of CASK in squamous cell malignancies, expression patterns of CASK were analyzed in Lung Squamous Cell Carcinoma (LUSC). Transcriptomic data were retrieved from UCSC Xena, which integrates the TCGA LUSC cohort and GTEx healthy lung samples [71]. In addition, proteomic data were retrieved from the CPTAC LUSC dataset [72,73].

### 5.18. Statistical Analysis

Values were presented as the mean ± S.E.M. of at least three independent experiments. Student’s *t*-test was performed to analyze the statistical significance of the differences, and a *p*-value < 0.05 was considered to be statistically significant.

## Data Availability

The data presented in this study are available on request from the corresponding author.

## Supplementary Materials

The following supporting information can be downloaded at: https://www.mdpi.com/article/10.3390/ijms27219909/s1.

## Abbreviations

The following abbreviations are used in this manuscript:

AREG	Amphiregulin
AKT	Protein kinase B
BSA	Bovine serum albumin
CASK	Calcium/calmodulin-dependent serine protein kinase
CDK2	Cyclin-dependent kinase 2
CENPM	Centromere protein M
CPTAC	Clinical Proteomic Tumor Analysis Consortium
DAPI	4′,6-diamidino-2-phenylindole
DE	Differential expression
DEG	Differentially expressed gene
DMEM	Dulbecco’s modified Eagle medium
DMSO	Dimethyl sulfoxide
E2A	E2A immunoglobulin enhancer binding factor
EGF	Epidermal growth factor
EGFR	Epidermal growth factor receptor
EREG	Epiregulin
ERK	Extracellular signal-regulated kinase
FBS	Fetal bovine serum
FDR	False discovery rate
GEO	Gene Expression Omnibus
GSEA	Gene set enrichment analysis
HaCaT	Human keratinocyte cell line
Id1	Inhibitor of DNA binding 1
JNK	c-Jun N-terminal kinase
LPEseq	Local-pooled-error sequence
LUSC	Lung squamous cell carcinoma
MAGUK	Membrane-associated guanylate kinase
MAPK	Mitogen-activated protein kinase
MTT	3-(4,5-dimethylthiazol-2-yl)-2,5-diphenyltetrazolium bromide
OXPHOS	Oxidative phosphorylation
PBS	Phosphate-buffered saline
PDZ	PSD-95/Dlg1/ZO-1
PI	Propidium iodide
p21	Cyclin-dependent kinase inhibitor 1A
p53	Tumor protein p53
qPCR	Quantitative polymerase chain reaction
Rb	Retinoblastoma protein
RNA-seq	RNA sequencing
SF	Serum-free medium
shRNA	Short hairpin RNA
siRNA	Small interfering RNA
TBS	Tris-buffered saline
TBST	Tris-buffered saline containing
TGF-α	Transforming growth factor-alpha
