# Supplementary material for: CASK Positively Regulates Keratinocyte Proliferation and Modulates the EGFR-ERK-p21 Signaling Axis
Source: Int J Mol Sci. 2026 Nov 5;27(21):9909. doi: 10.3390/ijms27219909 (PMC13763990; doi:10.3390/ijms27219909)
Supplement: Supplementary file 1 [file ijms-27-09909-s001.zip › ijms-4610138-supplementary.pdf]

Supplementary Table S1

Target mRNA	Forward primer (5’→3’)	Reverse primer (5’→3’)
p21	TGGAGACTCTCAGGGTCGAAA	GGCGTTTGGAGTGGTAGAAATC
p53	GCCCACTTCACCGTACTAACCA	GAAACTACCAACCCACCGACCA
EGFR	GACCTCCATGCCTTTGAGAA	GCTGACGACTGCAAGAGAAA
AREG	GCACCTGGAAGCAGTAACATGC	GGCAGCTATGGCTGCTAATGCA
EREG	CTTATCACAGTCGTCGGTTCCAC	GCCATTCAGACTTGCGGCAACT
TGF-α	GGTCCGAAAACACTGTGAGTGG	CAAACTCCTCCTCTGGGCTCTT
